# Supplementary material for: Angelica sinensis polysaccharide as potential protectants against recurrent spontaneous abortion: focus on autophagy regulation
Source: Front Med (Lausanne). 2025 Jan 15;12:1522503. doi: 10.3389/fmed.2025.1522503 (PMC11774876; doi:10.3389/fmed.2025.1522503)
Supplement: Supplementary material S1 — The main instruments used during the LC-MS process, along with their models/specifications and manufacturers. [file Supplementary_file_1.zip › Metabolomics sequencing data FC1.2/8.代谢通路分析/Exposure_Ctrl/all/KEGG/mmu04922.html]

KEGG PATHWAY: Glucagon signaling pathway - Mus musculus (house mouse)


# Glucagon signaling pathway - Mus musculus (house mouse)


[
Pathway menu
| Organism menu
| Pathway entry
| Show description
| Download
| Help
]

Glucagon is conventionally regarded as a counterregulatory hormone for insulin and plays a critical anti-hypoglycemic role by maintaining glucose homeostasis in both animals and humans. To increase blood glucose, glucagon promotes hepatic glucose output by increasing glycogenolysis and gluconeogenesis and by decreasing glycogenesis and glycolysis in a concerted fashion via multiple mechanisms. Glucagon also stimulates hepatic mitochondrial beta-oxidation to supply energy for glucose production. Glucagon performs its main effect via activation of adenylate cyclase. The adenylate-cyclase-derived cAMP activates protein kinase A (PKA), which then phosphorylates downstream targets, such as cAMP response element binding protein (CREB) and the bifunctional enzyme 6-phosphofructo-2-kinase/ fructose-2,6-bisphosphatase (one of the isoforms being PFK/FBPase 1, encoded by PFKFB1).


##### Option

Scale:


100%

Image resolution:


 High

##### Search

##### ID search

##### Color


KGML

Image (png) file 1x
